# Adsorption of dimethyl disulfide onto activated carbon cloth

**DOI:** 10.55730/1300-0527.3374

**Published:** 2022-02-23

**Authors:** Firdevs MERT SİVRİ, Numan HODA, Ayhan TOPUZ, Leyla BUDAMA AKPOLAT, Emrah EROĞLU

**Affiliations:** 1 Süleyman Demirel University, Department of Basic Pharmaceutical Sciences, Isparta, Turkey; 2 Akdeniz University, Department of Material Science and Engineering, Antalya, Turkey; 3 Akdeniz University, Department of Food Engineering, Antalya, Turkey; 4 Akdeniz University, Department of Chemistry, Antalya, Turkey

**Keywords:** Activated carbon cloth, adsorption, isotherms, adsorption kinetics, dimethyl disulfide

## Abstract

Dimethyl disulfide (DMDS) has a specific unpleasant odour and is profoundly toxic with an odour threshold of around 7–12 ppb. In this study, the removal of DMDS was investigated by adsorption on activated carbon cloth (ACC) in the gas phase. Kinetics and isotherm studies were performed. Adsorption kinetics followed by GC-MS and the data were processed using different models. When correlation coefficients (R^2^) of linear regression analysis are analyzed, it is seen that the concordance of experimental data to the pseudo-second-order equation is quite good. Isotherm data have been examined using Freundlich, Temkin and Langmuir models. The regression coefficient (R^2^) of data to fit the Langmuir model is 0.9993, which means that the fit is very good. The monolayer adsorption capacity (q_m_) of DMDS has been calculated as 118 mL.g^−1^ according to the Langmuir model.

## 1. Introduction

The application of adsorption for the removal of hazardous gases plays an important role in the prevention of health risks of human beings. These gasses cause cancer, throat irritation, liver damage, diminished lung function, respiratory illness, and mortality [1,2] For these reasons, the investigation into more effective reduction or disposal methods for VOCs is required [3,4]. The adsorption has been recognized as an effective and regenerative procedure for controlling VOC emissions, particularly at low concentrations [3–6]. Activated carbon (AC) is the most commonly utilized adsorption material for that goal because of its cost-effectiveness and its efficiency [7,8]. The powdered, granulated and cloth (fiber) forms of AC are widely used in industrial and research fields. Since the fiber/cloth form of AC has a high specific surface area and adsorption capacity etc., it has attracted much interest recently. Just as there are many applications and studies on liquid-phase adsorption of activated carbon cloth (ACC) [9,10], there is an increasing trend towards its application for gas phase adsorption due to its elasticity and faster adsorption kinetics. Another advantage of ACC in gas phase adsorption of VOCs was reported by Cal et al. (1997), who observed that water vapour adsorption had no significant effect on the adsorption of VOCs on ACC until the relative humidity increased above 50% (P/P_0_ > 0.5) [11]. In the literature, there are also some comparative studies for powdered, granular (GAC) and ACC to show better adsorption effectiveness of ACC. For example, Balanay et al., (2011) studied the comparison of the toluene adsorption of GAC and ACC types and their research exhibited that GAC, which has a higher BET surface area, has lower adsorption capacities than ACCs [12].

Dimethyl disulfide (DMDS) is a nonpolar, stable, and reduced sulfur compound that not only occurs in the natural environment, but is also emitted from the wood pulp industry [13], composting [14], oil refineries [15], landfills [16], and wastewater treatment plants [17]. DMDS vapours have a characteristic unpleasant odour and are highly toxic with an odour threshold of about 7–12 ppb [18]. It also has the highest insecticidal neurotoxicity in eukaryotic cells due to mitochondrial dysfunction [19,20].

The removal of DMDS has been investigated by many researchers using different kinds of adsorbents such as activated carbon, silica-gel, porous alumina, activated alumina and zeolite [21]. Among them, activated carbons seem to be the most effective and popular. The adsorption of DMDS vapours was investigated on two specimens of granular and two specimens of fibrous activated carbons, which had different surface areas and were related with different amounts of carbon-oxygen surface groups [22]. It was seen that the adsorption of DMDS on activated carbon depends upon the surface area, however is strongly affected by the existence of carbon-oxygen surface groups.

The goal of this study is to investigate the adsorption behavior of DMDS on the newly developed ACC and thus explore the possibility of removal DMDS from polluted air by performing kinetics and isotherm experiments in the gas phase.

## 2. Materials and methods

### 2.1. Materials

**Figure 1:**
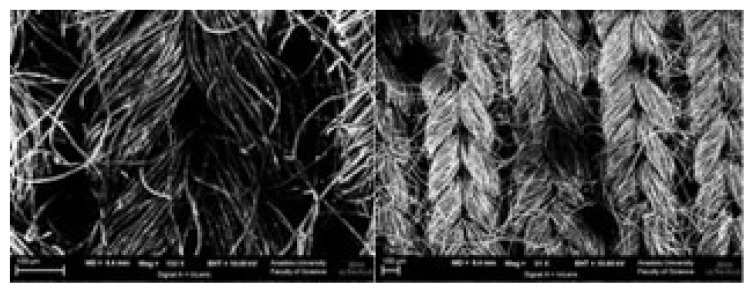
SEM pictures of ACC at different magnifications.

Nume Kimya (Antalya, Turkey) supplied ACC, encoded as GDSEL 651. GDSEL 651 is a cellulose-based ACC, which was activated with H_2_O. SEM picture of ACC used in the study is given in Figure 1. Dimethyl disulfide (DMDS) was purchased from Sigma. Merck provided hydrochloric acid, sodium carbonate, sodium bicarbonate, sodium hydroxide and nitric acid. In preparation of all solutions, deionized water was used.

### 2.2. Treatment of ACC

Warm distilled water was used to wash ACC Sample (5 L at 60 °C) to remove impurities that might be present during the preparation of ACC. During washing, N_2_ was introduced into the wash water and the water used was changed until the conductivity of the water remained unchanged. After the washing step, the ACC sample was dried at 120 °C for 6h in a vacuum oven.

### 2.3. Characterization of ACC

The experiment of nitrogen adsorption was performed for identification of the surface properties of ACC as described below. Degassing of the ACC samples was carried out at 130 °C under vacuum (up to 10^−6^ torr) for 12 h, and nitrogen adsorption data were collected using the Quantachrome Autosorb -1-C/MS equipment. The BET-specific surface area (S_BET_) of the ACC was calculated over a relative pressure ranging from 10^−6^ to 1 using the software of the apparatus.

The pH_pzc_ value of washed ACC was determined using the method described by Babic et al [23]. 20 mL of 0.01 M NaNO_3_solutions with different initial pH values adjusted by adding NaOH or HNO_3_ solutions were prepared. Constant weighted ACC samples were placed into solutions and shaken for 24 h. After that, the equilibrium concentrations of H^+^ and OH^−^ ions in each sample solution were determined using a pH meter. Then, adsorbed amounts of OH^−^ and H^+^ ions onto ACC samples were calculated by subtracting the final measured concentrations of H^+^ and OH^−^ ions from the initial concentrations. pH_pzc_ value for ACC was taken as the unchanged pH at which initial and final pH measurements.

The surface functional groups of ACC were determined by the procedure given by Boehm [24] and FTIR-ATR spectroscopy. In the titration procedure, 0.05 g of ACC sample was placed into acidic and basic solutions of HCl, NaOH, Na_2_CO_3_, and NaHCO_3_ which were prepared previously. The solutions were stirred for 48 h and samples were taken out from solution by tweezers. Aliquots of the solution (20 mL) were titrated using 0.1 M HCl or 0,1 M NaOH depending on whether the acidic or basic oxygen-containing functional groups were determined. According to Boehm’s method, NaOH neutralizes carboxylic, phenolic and lactonic groups, Na_2_CO_3_ neutralizes carboxylic and phenolic groups, NaHCO_3_ only neutralizes carboxylic groups.

Fourier Transform Infrared Spectroscopy-Attenuated Total Reflectance (FTIR-ATR) was performed using a BRUKER, Tensor 27 with a scan range of 4000–400 cm^−1^.

### 2.4. Adsorption cell

**Figure 2:**
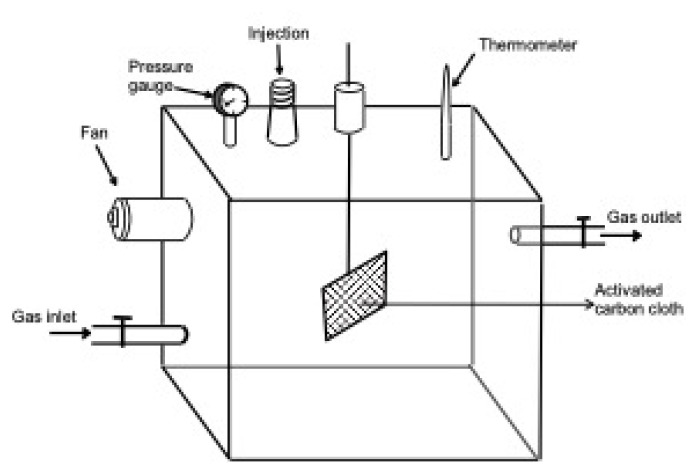
The schematic diagram of home built static gas adsorption apparatus.

The adsorption investigations were performed via the utilization of a custom home-built cell. The detailed diagram of this cell is given in Figure 2. The cell has a volume of 8.7 L, equipped with a thermometer, a fan, a pressure gauge, a sample holder, injection hole (capped with a septum), and gas inlet/outlet.

### 2.5. Adsorption kinetics measurements with GC-MS

Shimadzu GCMS-QP2010 Plus model GC-MS was used for estimating DMDS in the cell. ACC sample was placed into the sample holder of the cell. After sealing, the cell was vacuumed and filled with dry air until one atmospheric pressure is reached in the cell. A certain amount of sample (DMDS) was injected into the cell and the fan was operated. Preliminary tests without placing ACC sample in the cell showed that DMDS equilibrated (homogeneously distributed in the cell) within 5 min in the gas phase. In the adsorption kinetics experiments, the initial concentration of DMDS was kept constant but the amount of ACC samples placed into the cell was varied. For each ACC sample, 100 μL gas was taken and injected into GC-MS in the first 15 min, and then every 30 min to determine the remaining DMDS in the cell.

### 2.6. Determination of adsorption isotherms

DMDS adsorption isotherms on ACC were derived using batch analysis. The pieces of ACC with varying masses were placed in the cell and allowed to equilibrate at a constant initial concentration of 11.5 ml.L^−1^ DMDS at 25 °C for 24 h. Before the adsorption isotherms experiments, some preliminary tests were performed and it was found that the DMDS concentration remained unchanged after 3 h of contact with the ACC. In the adsorption isotherm experiments, the duration of adsorption was held for 24 h to get full equilibration for DMDS. For all ACC pieces worked, the DMDS amount adsorbed per unit mass of ACC, q_e_, a parameter needed for derivation of isotherm, was calculated using equation (1),


(1) $${\text{q}}_{\text{e}}=\frac{\text{V}.({\text{C}}_{0}-{\text{C}}_{\text{e}})}{\text{m}}$$

V: DMDS volume (volume of the cell, L)
C_0_: The preliminary concentrations of DMDS (mL.L^−1^)
C_e_: The equilibrium concentrations of DMDS (mL.L^−1^)
m: The mass of ACC (g).

## 3. Results and discussion

### 3.1. Characteristics of ACC

**Figure 3:**
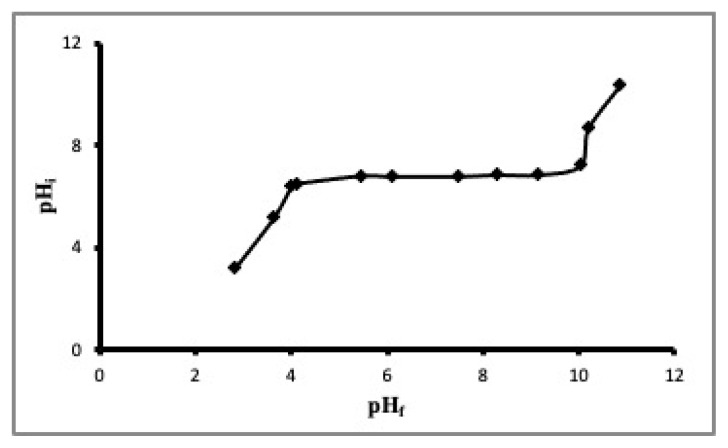
The result of pHpzc measurements of ACC.

Brunauer, Emmet, and Teller method was employed to calculate the specific surface area of ACC (S_BET_), using the linear section of the nitrogen adsorption isotherm. The S_BET_ was found to be 500 m^2^.g^−1^ with a total micropore area of 498 m^2^.g^−1^. According to surface area measurement results, it can be concluded that ACC has a totally microporous structure. The pH_pzc_ value of ACC, which is used in this study, was 6.8 ± 0.06 (Figure 3). The surface oxygen containing functional groups were determined as 3.8 ± 0.05 meq/g (total acidic groups), 4.1 ± 0.04 meq/g (total basic groups), 2.0 ± 0.04 meq/g (carboxylic groups), 0.4 ± 0.003 meq/g (lactonic groups), and 1.4 ± 0.05 meq/g (phenolic groups).

**Figure 4:**
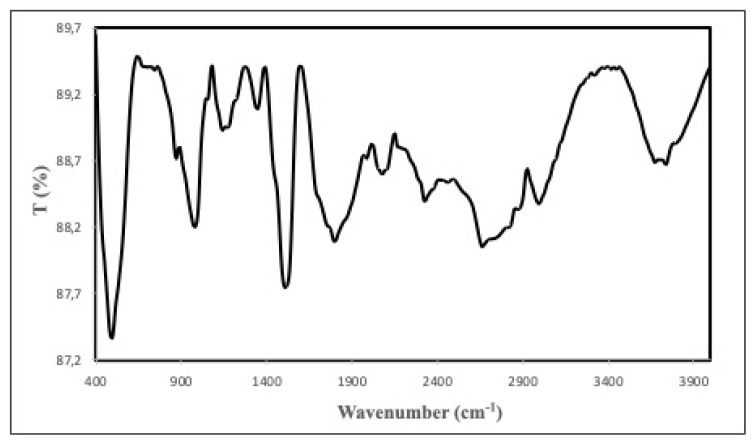
The FTIR-ATR spectrum of ACC.

The FTIR-ATR spectroscopy was also used to identify the functional groups on the surface of ACC studied and the spectrum is given in Figure 4. In the spectrum, the broad band around at 3747 cm^−1^ can be attributed to the stretching vibration of O-H groups. It may also come from adsorbed water onto ACC. The peak around at 3000 cm^−1^ is assigned to aromatic C-H strethcing vibrations [25]. The peaks observed at 2883 and 2829 cm^−1^ can be assigned to the stretching and scissoring of C–H bonds [26]. While C-H stretching vibration is observed at around 2330 cm^−1^, the C–C triple bond stretching is appeared at the peak around 2082 cm^−1^ [27,28]. The existence of shoulders at 1758 and 1698 cm^−1^ corresponds to carbonyl groups C=O or COOH) or C=O in ester groups [29]. The peak at 1512 cm^−1^ may be attributed to C=C vibrations in the aromatic groups [30] The band at 1350 cm^−1^ can be ascribed to vibrations of the carboxyl groups (-COO-) on the surface of ACC [30,31]. The bands at 1181, 1144, and 1058 may correspond to the stretching of the C-O group from alcohol, acids, phenols, ethers, and/or ester functional groups [32]. While the peaks at 872 and 493 cm^−1^ represent aromatic C–H out-of-plane bend, the peak at 982 cm^−1^ represents vinyl C-H out-of-plane bend [25,33,34]. Both the Boehm method and FTIR-ATR analysis performed on ACC studied confirmed functional groups such as -OH, C-O, C=O, and -COOH in the structure of ACC, which play an important role adsorption process.

### 3.2. Adsorption kinetics of DMDS

**Figure 5:**
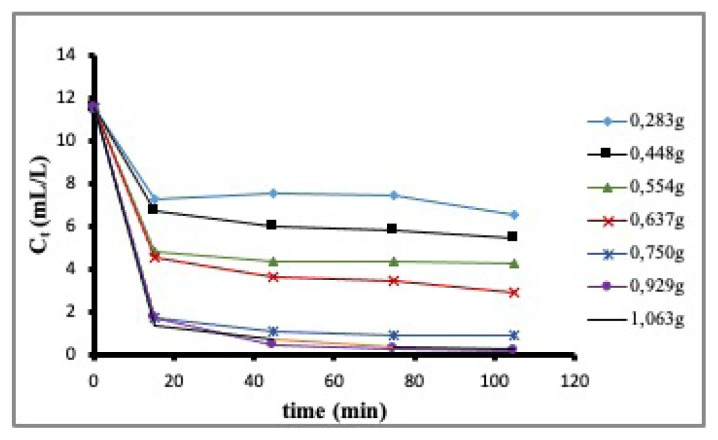
Kinetics of adsorption of DMDS onto ACC with varying masses.

Adsorption kinetics of DMDS onto ACC was followed using GC-MS method outlined earlier. The data of chromatographic was converted to data of concentration using corresponding calibration and later plotted as a function of time in Figure 5 for different amounts of ACC samples with the constant initial concentration of 11.5 ppm of DMDS. Adsorption was followed during the first 105 min by measuring the concentration in the cell at the first 15 min and then at 30 min intervals. As observed in Figure 5, the amount of DMDS adsorbed onto ACC increases as the amount of ACC is increased, but when the amount of ACC exceeds ~0.9 g, there is no change in the kinetics of adsorption. Therefore, kinetic calculations were evaluated using the data for 1.063 g ACC assuming that equilibrium is reached in the first 105 min.

To examine the behavior of the adsorption process of DMDS onto ACC pseudo-first-order [35] and pseudo-second-order [36] models were applied to kinetic data of adsorption. The pseudo-first-order model is linearized with the following equation;


(2) ln(qe-qt)=lnqe-k1t

q_e_: DMDS masses adsorbed at equilibrium (mL.g^−1^)
q_t_: DMDS masses adsorbed at time (mL.g^−1^)
k_1_: the rate constant (min^−1^).

For the ACC sample, the k_1_ value is derived from the slope of the curve ln (q_e_−q_t_) against t. The q_t_ value is determined using the following formula:


(3) $${\text{q}}_{\text{t}}=\frac{\text{V}.({\text{C}}_{\text{o}}-{\text{C}}_{\text{t}})}{\text{m}}$$

V: The DMDS volume (volume of the cell)
m: The mass of ACC samples.

When the linear regression analysis was evaluated according to the linearized version of the first-order pseudo-model, R^2^ was found as 0.9063.

It is possible to state the linearized pseudo-second-order model as follows,


(4) $$\frac{\text{t}}{{\text{q}}_{\text{t}}}=\frac{1}{{\text{k}}_{2}{\text{q}}_{\text{e}}^{2}}+\frac{1}{{\text{q}}_{\text{e}}}\;\text{t}$$

k_2_: the second-order rate constant (g.mL^−1^.min^−1^).

The linear plot of t/qt against t yielded the values of k_2_ and q_e_ (theoretical value). The (R^2^) value for the pseudo-second-order was found as 0.9998. When the correlation coefficients (R^2^) obtained from the linear regression analysis are investigated, the fit of the experimental data to the pseudo-second-order equation seems to be good. Therefore, it is possible to infer that the pseudo-second-order model describes the adsorption kinetics of DMDS onto ACC. Also, the pseudo-second-order model showed that the theoretical value of equilibrium adsorption capacity, q_e_ (cal), which is calculated from the model as 92.15 mL.g^−1^, was close to the experimental q_e_(exp) value, which is 94.34 mL.g^−1^.

### 3.3. Adsorption isotherms

Different amounts of ACC samples at a fixed concentration in the adsorption cell were employed to define adsorption isotherms of DMDS at 25 °C. To evaluate for the fitness of adsorption isotherm data obtained in the experiments, Freundlich, Temkin, and Langmuir isotherm models were used. The fitness of experimental data to these isotherm models and their parameters obtained are given in Table.

The isotherm of Langmuir is based on the theory that adsorption occurs at specific homogeneous regions in the adsorbent. The Langmuir isotherm assumes that there is no significant interaction between the adsorbed types and the adsorbent becomes saturated after a layer of adsorbed types is formed on the adsorbent surface. The equation of Langmuir isotherm as follows,


(5) $$\frac{C_{e}}{{\text{q}}_{\text{e}}}=\frac{1}{\text{b}q_{m}}+\frac{C_{e}}{{\text{q}}_{\text{m}}}$$

q_e_: The mass of solute adsorbed per unit mass of adsorbent (mL.g^−1^)
C_e_: The equilibrium concentration of solute in the gas phase (mL.L^−1^)
q_m_: Adsorption capacity for complete coverage of the surface with a monolayer (mL.g^−1^)
b: A constant associated to the heat of adsorption (L.mL^−1^).

**Figure 6:**
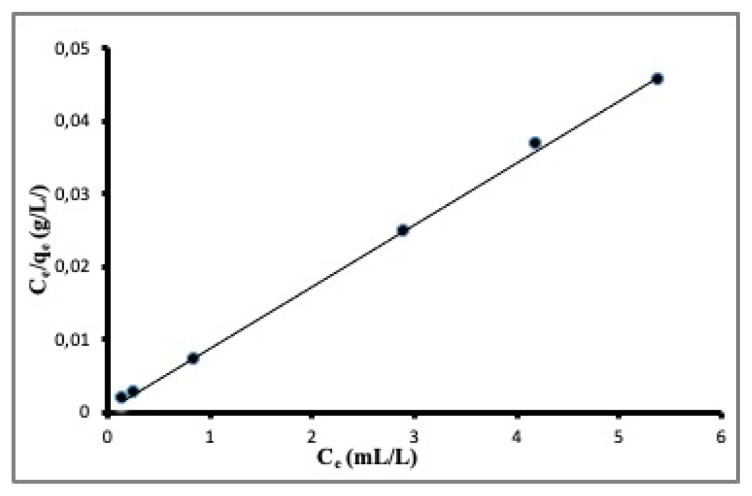
The fit of experimental data to the Langmuir model.

The constants q_m_ and b can be calculated from the slope and intercept of the plot of C_e_/q_e_ versus C_e_. The plot for experimental data with linearized Langmuir equation is shown in Figure 6. The regression coefficient (R^2^) for the fit of the Langmuir model is 0.9993, which means that the fit is excellent. Langmuir model has been used to calculate q_m_ for DMDS and found to be 118 mL g^−1^.

The separation factor (R_L_) is commonly used to estimate the efficiency of the adsorption process [37]. (R_L_) was determined utilizing formula below using the Langmuir parameter “b” in equation (6);


(6) $$R_{L}=\frac{1}{1+{\text{bC}}_{0}}$$

b: The constant of Langmuir (L.mL^−1^)
C_0_: The preliminary concentration (mL.L^−1^).

Depending on the value of R_L_, the Langmuir isotherm is classified as unfavorable, favorable, linear, and irreversible. When the R_L_ value is between 0 and 1, isotherm is considered to be favorable [37]. Since R_L_ value was determined as 0.0235, the isotherm is considered to be favorable.

**Figure 7:**
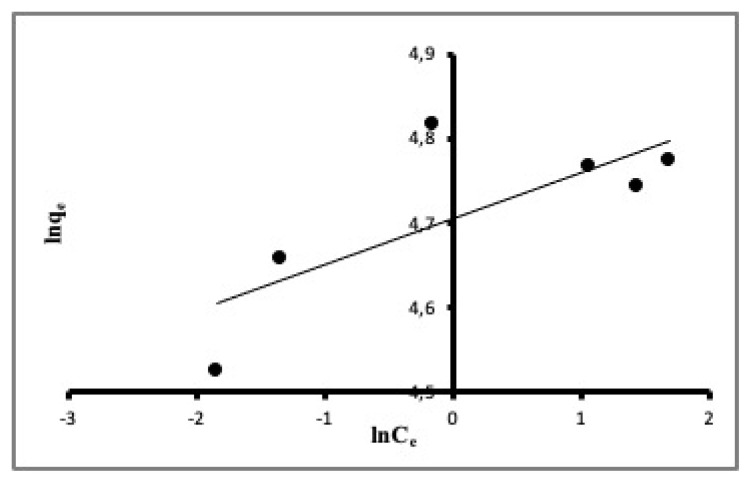
The fit of experimental data to the Freundlich model.

The Freundlich isotherm model supposed that the adsorption on the surface is multilayered and heterogeneous due to the uneven distribution of active sites. The linearized of the Freundlich isotherm model can be stated as the following,


(7) $${\text{lnq}}_{\text{e}}=\text{lnK}+\frac{1}{\text{n}}\text{ln }{\text{C}}_{\text{e}}$$

where q_e_ and C_e_ can be defined as in the Langmuir equation. K is Freundlich constant and 1/n is an empirical constant known as heterogeneity factor which is related to the surface heterogeneity. The constants K and 1/n are calculated using the slope and intercept of the equation plot, respectively. According to McKay and Ho (1999), the heterogeneity factor ranges from 0 to 1; the more heterogeneous the surface, the nearer the value of 1/n to 0 [36]. The plot for Freundlich’s linearized isotherm equation of the experimental data is shown in Figure 7. As seen in Figure 7 and from the R^2^ value of the plot (0.5860), the fitting of the experimental data to the Freundlich model is not good.

The Temkin model, which is the third one of the isotherms, tested for the suitability of DMDS adsorption data. The Temkin model related to indirect adsorbent-adsorbate interactions on adsorption isotherms and assuming the heat of adsorption decreases linearly as coverage of adsorbate molecules increases and formulated as [38];


(8) $${\text{q}}_{\text{e}}={\text{B}}_{\text{T}}\;{\text{lnA}}_{\text{T}}+{\text{B}}_{\text{T}}\;{\text{lnC}}_{\text{e}}$$

B_T_: A constant associated to the heat of sorption (equal to RT/b)
A_T_: Temkin’s isothermal equilibrium binding constant and it is possible to determine the constants using the slope and intercept.

**Figure 8:**
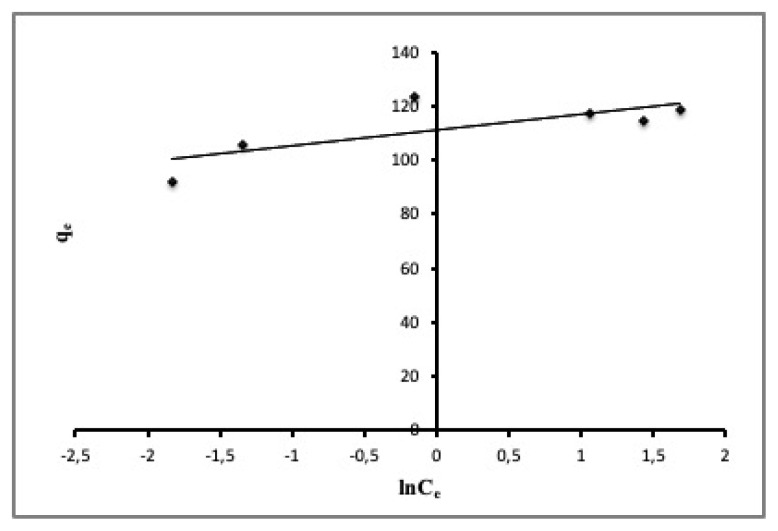
The fit of experimental data to the Temkin model.

Linear regression analysis of adsorption data to the Temkin isotherm model is shown in Figure 8. As can be seen the distribution of data in Figure 8 and regarding with R^2^ which was found to be 0.5741, meaning that it is very poor for fitting the adsorption data to the Temkin isotherm model.

Adsorption of DMDS was also studied in dynamic phase by Vega et al. (2013) using steam-activated RB3 charcoal (granulated) from Norit RB3 charcoal having the BET surface area of 927 m^2^.g^−1^ and the adsorption capacity for DMDS has been determined as 108 mg.g^−1^ [39]. Obviously, the adsorption capacity of ACC studied was higher than RB3 charcoal’s adsorption capacity, although RB3 has almost two-fold higher surface area. ACC has a higher microporous area providing more accessibility from external to inner surfaces of the fiber. So, adsorptive molecules can reach adsorption sites which are on the wall of micropores without any diffusional resistance unlike in macropores. The adsorption experiments were performed in a dry medium, therefore, the adsorption of DMDS onto ACC can be considered as the result of hydrogen bonding between the carbon-oxygen surface groups on ACC and the thiol of DMDS [22].

## 4. Conclusions

In this study, adsorption kinetics and equilibrium isotherms of adsorption of DMDS onto ACC were investigated. It was found that the pseudo-second-order model describes the adsorption kinetics of DMDS onto ACC. The pseudo-second-order model showed that the theoretical value of equilibrium adsorption capacity, q_e_ (cal), which is calculated from the model as 92.15 mL.g^−1^, was close to the experimental q_e_ (exp) value, which is 94.34 mL.g^−1^. Isotherm data have been examined using Freundlich, Temkin and Langmuir models. The regression coefficient (R^2^) of data to fit the Langmuir model is 0.9993, proving that the fit is high enough to conclude that the adsorption process of DMDS onto ACC follows the Langmuir model. The monolayer adsorption capacity (q_m_) of DMDS has been calculated as 118 mL.g^−1^ according to the Langmuir model.

Another conclusion that can be drawn from the results is that the adsorption of DMDS on activated carbons is directly related to the microporosity and form of the activated carbons. Although ACC studied has a BET surface area of 500 m^2^.g^−1^, it has almost the same adsorption capacity as GAC having a BET surface area of 927 m^2^.g^−1^. But here it should be stated that ACC is more microporous than GAC. Considering the advantages of ACCs over GACs, ACC can have the potential for use as adsorbents in thinner and lighter disposable respirators or masks for protection against DMDS, and probably other VOCs.

In future studies, adsorption DMDS other VOC’s onto ACC will be investigated in different relative humidities to determine its capacity and dynamic phase adsorption capacity with varying flow rate for real respiratory applications.

## Figures and Tables

**Table:** The fitness of experimental data to Freundlich, Temkin, and Langmuir isotherm models and their parameters.

**ACC**	**Langmuir**			**Freundlich**			**Temkin**		
	q_m_ (mL.g^−^^1^)	b (L.mL^−^^1^)	R^2^	K(mL^1^^−^^(1/n)^ L^1/n^ g^−^^1^)	1/n	R^2^	A_T_ (L.mg^−^^1^)	B	R^2^
	118	42.37	0.9993	110.52	0.0549	0.5860	4.12 × 10^5^	9.0572	0.5741
